# Robotic Anxiety—Parents’ Perception of Robot-Assisted Pediatric Surgery

**DOI:** 10.3390/children9030399

**Published:** 2022-03-11

**Authors:** Elisabeth Ammer, Laura Sophie Mandt, Isabelle Christine Silbersdorff, Fritz Kahl, York Hagmayer

**Affiliations:** 1 Department of General, Visceral, and Pediatric Surgery, University Medical Center Goettingen, 37075 Goettingen, Germany; fritz.kahl@med.uni-goettingen.de; 2 Georg-Elias-Mueller Institute for Psychology, University Goettingen, 37073 Goettingen, Germany; laurasophie.mandt@stud.uni-goettingen.de (L.S.M.); i.silbersdorff@stud.uni-goettingen.de (I.C.S.); york.hagmayer@bio.uni-goettingen.de (Y.H.)

**Keywords:** robot-assisted surgery, laparoscopic surgery, pediatric surgery, parents, behavioral intention, operation method, anxiety, risk perception, benefit perception, child’s age

## Abstract

In contrast to many other countries, robot-assisted (RA) pediatric surgery is not yet very common in Germany. Although the first pediatric RA intervention was published in 2001, RA pediatric surgery is still perceived as a “new technology”. As a consequence, little is known about parents’ perception of this operation method. In this study, we analyzed parents‘ intention to let their child undergo RA and laparoscopic (LA) surgery. Two subsamples (online and at the University Medical Center Goettingen) received a questionnaire addressing attitude towards RA and LA pediatric surgery with the help of a case example. Results showed that parents had a higher intention to consent to LA surgery. Perceiving more benefits, assuming a positive attitude of the social environment, and feeling less anxiety increased intention. A mediation analysis indicated that the type of surgery affected intentions through assumed attitude of the social environment. Exploratory analyses showed that the perception of risks and anxiety reduced intention for only RA surgery. These findings should be considered in preoperational discussions with parents. Anxiety and perceived risks should especially be addressed in order to encounter hesitancy.

## 1. Introduction

Robotic surgery has long found its place not only in urology, gynecology, and visceral surgery but also in pediatric surgery. Since the first published robot-assisted (RA) pediatric intervention in 2001, many more cases have followed [1]. With the US leading the way, many European countries have caught up and successfully performed various surgeries. They range from pyeloplasties or bladder augmentations to hepatobiliary surgery and thoracoscopic interventions. This upward trend is apparent by the fact that 40% of pyeloplasties carried out in the US in 2015 were RA [2,3,4]. Over the years, not only has the range of RA interventions broadened but the use in very small children has also increased [5,6]. In accordance with this development, more publications on RA pediatric surgery have been released in recent years [7,8]. Various publications have emphasized that RA surgery in a pediatric population is not only feasible and safe but also results in the same or even better outcome with a similar complication rate as laparoscopic or open procedures [9,10]. Surgeons applying RA surgery value advantages such as 3D view of the operation site, tremor-free hand movement, improved dexterity, and ergonomics [4,11], while patients profit from decreased operative time, reduced pain, and reduced length of postoperative hospital stay [12]. However, RA pediatric surgery also has disadvantages, including lack of specific instruments, limitations depending on the size of the child, no haptic feedback, and high costs [4,13,14].

Unlike the multitude of publications on implementation and feasibility of RA pediatric surgery, there have been few studies focusing on public perception of robotic surgery, let alone the perception of parents. Although many people have heard about RA surgery, they only have a rough or even wrong idea of how it actually works [15]. On the one hand, surgeons offering RA surgery are perceived as more skilled than nonrobotic surgeons. On the other hand, one often-expressed belief is that the surgical robot is acting on its own accord without the surgeon controlling it. Another misassumption is that RA surgery is similar to open or scarless/laser surgery. Many people are afraid that the robot might malfunction during surgery [15,16,17]. Although a multitude of new technological developments affect our everyday life, RA surgery is sometimes perceived as threatening [18].

Jank et al. [19] published a study examining parents whose children had undergone robotic cochlear implantation. Apart from that, there are no studies to date on the perception, attitude, or possible anxiety of parents towards RA pediatric surgery. With this study, we try to bring light into the darkness in this area. Our objectives were to (a) investigate which variables affect parents’ intention to let their child undergo RA surgery, (b) analyze possible mediation and moderation of the different parameters, and (c) draw conclusions on how to handle parents whose children may undergo RA pediatric surgery.

## 2. Methods and Materials

**Figure 1:**
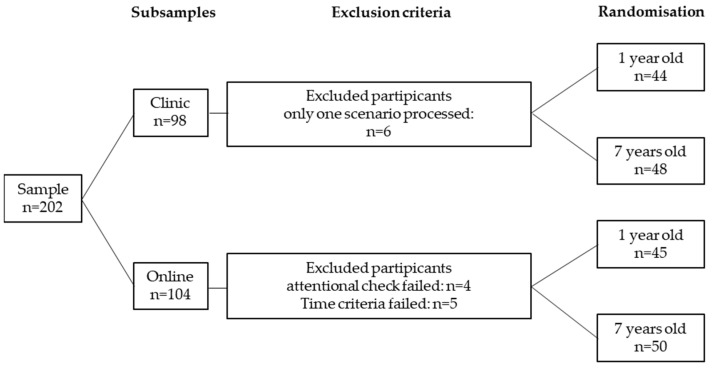
Composition of the sample. Two test sets were conducted: one online using an online participant recruitment platform (Prolific) and one among parents whose children were treated at the University Medical Center Goettingen (pediatric surgery and pediatric orthopedics). The distributed questionnaires were randomized regarding the age of the child presented in the scenario. Questionnaires with only one scenario being processed (clinical arm) or participants who failed the attention check or the time criteria (online arm) were excluded.

The study was a survey based on a structured questionnaire (see Appendix A) distributed among parents of young children in two different settings. The first subsample consisted of 98 parents whose children were patients at the Department of General, Visceral, and Pediatric Surgery or Pediatric Orthopedics (Department of Trauma Surgery, Plastic Surgery and Orthopedics) at the University Medical Center Goettingen. Their children were treated at either the outpatient department or an inpatient setting. Questionnaires could be either inserted into a voting box or filled out at home and returned by a stamped return envelope. The second subsample consisted of 104 German parents recruited via Prolific (prolific.org) who had at least one child born between 2006 and 2021. Parents in this subsample were paid for their time in accordance to the German minimum wage. Exclusion criteria for both subsamples were unwillingness to participate in the study or insufficient German language proficiency as the questionnaire was in German. Participants in the online subsample who failed an attention check (*n* = 4) or completed the questionnaire in less than half of the usual time (*n* = 5) were excluded because they obviously did not carefully attend to the questionnaire. In the clinical subsample, participants who responded to only one-half of the case scenario or less than half of the questionnaire overall were excluded (*n* = 6). This led to a final sample size of 187 participants (93% of the initial sample) (Figure 1).

**Figure 2:**
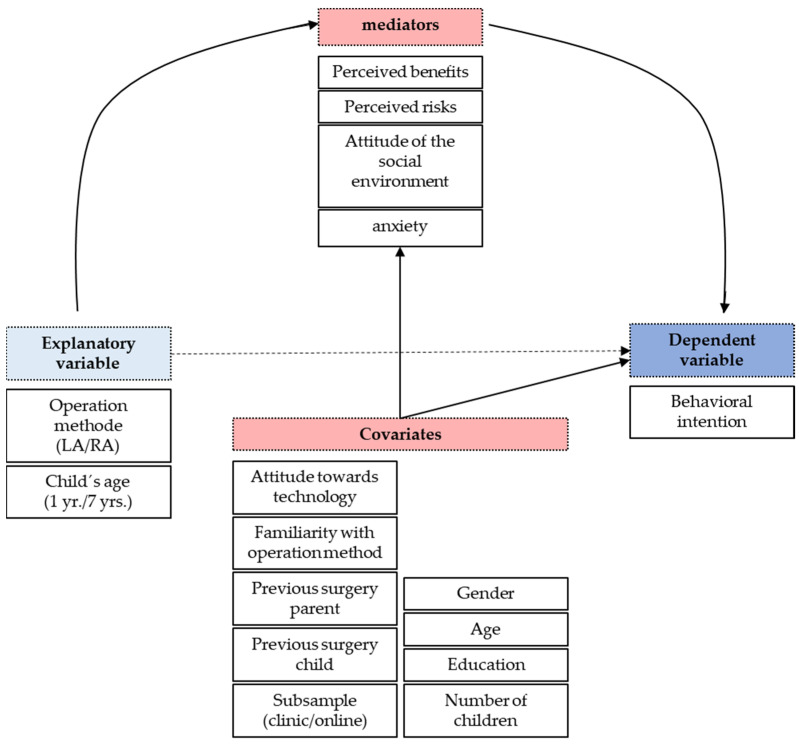
Theoretical model underlying the present research. It explains parents’ intention to consent to a surgery for their child. According to the model, the intention depends on the perceived benefits and risks, the emotional reaction (i.e., the anxiety experienced), and the attitudes of other relevant people. These factors in turn were assumed to be affected by the operation method and the age of the affected child. Attitude towards technology, familiarity with the operation method, previous surgery concerning the parent or a child, gender, age, level of education, and number of children served as covariates.

The study was based on the Unified Theory of Acceptance and Use of Technology (UTAUT) [20], which is a very well-established theory that allows people’s intention to use a new technology to be assessed and explained. According to the theory, there are three main factors: the expected performance (expected benefits and risks of a certain type of surgery), the effort expectancy (the perceived accessibility of a type of surgery), and the social influence (the assumed attitude of other relevant people). Following previous findings that emotional reactions may also be crucial [21], we added anxiety as a further factor. The theory also acknowledges that there might be numerous covariates that may also affect intention. Following this theoretical model, we expected the type of surgery (RA vs. laparoscopic (LA)) and the age of the affected child to influence the main factors and in turn the intention to have an operation with a certain type of surgery being performed. Figure 2 shows the theoretical model underlying the present research and the covariates we considered. Note that we excluded the effort expectancy factor from the model as both types of surgery were obviously available to parents in the clinical sample, while participants in the online sample indicated that they could not answer this question.

To investigate parents’ intention to use RA surgery, we presented them with a case scenario. It described a planned pyeloplasty for either a 1-year-old or a 7-year-old child. Pyeloplasty was chosen as it is a common and often-performed operation with robotic assistance [22]. Parents were asked to imagine that it was their child who needed to be operated on. In two consultations, an LA or RA operation was recommended to them, pointing out the same potential complications. A graphic representation was shown to illustrate the procedures. After each recommendation, parents were inquired about their emotional reaction to the recommended operation, the perceived benefits and risks, the assumed attitudes of other relevant people, and their intention to have the operation performed. Importantly, the order of the recommended operation method (RA vs. LA) was randomized.

The questionnaire was subdivided into three sections: a general part, the case scenario, and a sociodemographic section. Overall, it contained 60 items and took about 15 min to complete.

Unless mentioned otherwise, all items were rated on a five-point Likert scale. In Section 1, familiarity with RA/LA interventions was assessed by the familiarity scale from Anania et al. [21]. Attitude towards technology was assessed based on Wilkowska et al. [23].

In Section 2, the case scenario was presented, and the variables and covariates shown in Figure 2 were assessed after each operation procedure. Basic emotional reactions according to Ekman und Friesen [24] were inquired (anger, disgust, fear, happiness, sadness, and surprise) using a 10-step Likert scale. As we were interested in anxiety, we focused on fear. Expected benefits and risks were inquired with three items each, with the benefits taken from Wilkowska et al. [23]. Attitude of the social environment towards the operation method was inquired with three items asking about friends, family, and medical staff (6-step Likert scale). Medical staff were included because previous research has shown its relevance [19]. Behavioral intention (dependent variable) was assessed with three items again following Anania et al. [21]. Note that all items were inquired for an LA operation as well as an RA surgery.

In Section 3, demographic data were collected, including age, gender, number of children, highest graduation, previous surgery affecting participants or their child (under general anesthesia and/or RA), RA surgery planned for their child, and space for free text.

The study period was from March to May 2021. The questionnaire was tested in advance with a student sample to ensure that questions were clear and scales had an acceptable internal consistency.

### Statistical Analysis

Statistical analyses were performed using R version 4.0.5 [25]. Both subsamples were analyzed together as there were no significant differences with respect to the main variables, apart from a significantly higher anxiety in the online sample. Mean and standard deviation were computed as descriptive statistics for continuous variables, while frequencies and percentages were used for categorical variables. For the variables perceived benefits, perceived risks, social influence, and behavioral intention, the mean of the respective items was calculated per person before averaging across participants. To analyze potential differences between operation methods with respect to behavioral intentions, perceived benefits and risks, attitude of social environment, and emotional reactions, paired *t*-tests were computed. Due to multiple testing, a Bonferroni correction was applied and the significance levels were adjusted.

According to our theoretical model shown in Figure 2, perceived benefits and risks, social influence, and anxiety are mediators of the influence of the operation method on parents’ intention. Therefore, we conducted a mediation analysis based on Baron and Kenny [26]. For this, we computed linear mixed models that included covariates being significantly related to behavioral intention. To account for the fact that each participant judged both operation methods, participant-specific random intercepts were included in the models. To further test the theoretical model, we also investigated whether the operation method moderated the influence of perceived benefits and risks, social influence, and anxiety on parents’ intention.

## 3. Results

The final sample consisted of 187 parents (92 in the clinical setting and 95 from the online survey). Demographic data are shown in Table 1. The majority of participants were female (117; 62.5%), the mean age was 37.5 years (SD 7.82), and the average number of children was 1.96 (SD 1.16). The majority of participants in both subsamples had a high school diploma or a university degree. Most parents (74.4% overall) had undergone surgery in the past, but very few (1.6%) had robot-assisted (RA) surgery. Fewer children had surgery in the past (38.0%), but the percentage of RA surgery was higher (7.5%). For 2.1% of the children, an RA operation was planned. Unsurprisingly, children in the clinical setting had a higher rate of previous surgeries. Overall, attitude towards technology was rather positive. It was significantly higher in the online sample (*p* = 0.002), as one might have expected.

Table 2 shows the mean results for the main variables for laparoscopic (LA) and robot-assisted (RA) surgery. Apart from anxiety, there were no significant differences with respect to the age of the child in the scenario, so overall means (and SD) are presented. Participants reported higher anxiety levels regarding RA surgery. Anxiety was higher for the 1-year-old child compared to the 7-year-old child (mean of 6.5 for the 1-year old child vs. mean of 5.81 for the 7-years old child; data not shown in Table 2). The perceived benefit of LA surgery was higher, while there were no differences in perceived risks. Participants also believed their social environment would favor LA surgery over RA operations. The intention to let their child be operated on by a specific method was higher for LA surgery (3.97 vs. 3.63). Regarding potential covariates, familiarity with LA was significantly higher than with RA (2.60 vs. 2.27, *p* < 0.001).

For the mediation analysis, we first computed a model with covariates predicting behavioral intention. It turned out that familiarity with the procedure and attitude towards technology were significantly associated with intention to perform the procedure. Having a child who had surgery was marginally significant. A model with these three variables explained 11% of the variance in parents’ intentions (Model 1 in Table 3).

In the next step, the effect of the two manipulated variables on behavioral intention was examined, controlling for the covariates from Model 1 (see Model 2 in Table 3). Here, the operation method (LA vs. RA) had a significant effect on the intention, whereas the age of the affected child did not. The intention to consent declined for RA in comparison to LA.

In the following step, we examined whether the operation method and age of the affected child had an effect on anxiety, perceived benefits and risks, and the assumed attitude of the social environment, again controlling for the covariates from Model 1 (see Table 3: Models 3a–d). It turned out that anxiety was negatively predicted by having a child who had previously undergone an operation with anesthesia. This means a previous surgery reduced the level of anxiety. Perceived benefits were significantly predicted by attitude towards technology. Operation method and having a child operated on before did not reach significance when the number of statistical tests was taken into account. Perceived risks were significantly predicted by attitude towards technology. The assumed attitude of the social environment was also predicted by attitude towards technology, having a child operated on before, and operation method. Participants assumed relevant other people to have a more positive attitude towards laparoscopic surgery. The results of these four models show that only the assumed attitude of the social environment could be a mediator of the effect of the operation method on behavioral intention.

**Figure 3:**
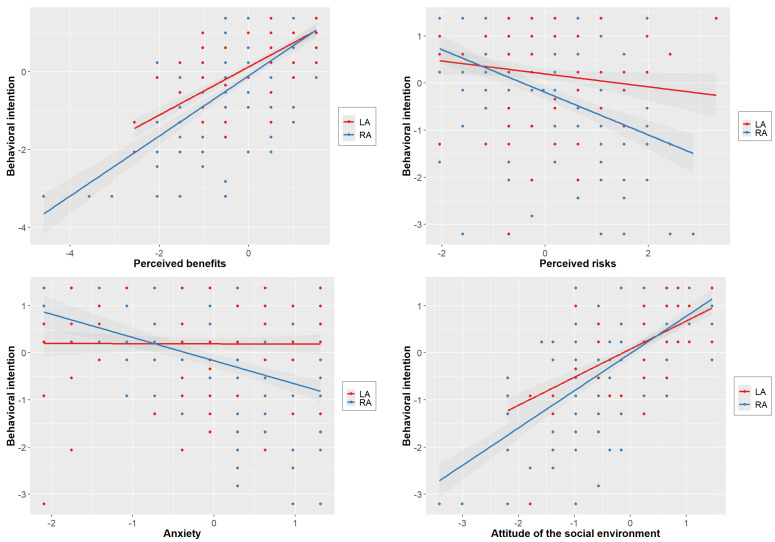
Influence of perceived benefits, perceived risks, assumed attitude of the social environment, and anxiety on behavioral intention to consent to a laparoscopic (LA) or robot-assisted (RA) surgery. Perceived benefits and attitude of the social environment showed a positive relationship to intention, anxiety, and perceived risks in an ordinal interaction.

Finally, a model including the three covariates, manipulated variables, and potentials mediators as predictors of behavioral intentions was computed (see Model 4 in Table 3). Figure 3 illustrates the relationship between behavioral intention and the four main predictive variables separated for LA and RA. Anxiety, perceived benefits, and assumed attitude of social environment were significant predictors of the intention to consent, while operation method was no longer predictive. Thus, the attitude of the social environment mediated the effect of operation method on intention. Interestingly, the results also showed that the influence of attitude towards technology was mediated by perceived benefits and risks as well as the attitude of the social environment. Participants, who had a positive attitude towards technological advances saw more benefits and less risks and assumed others to be more in favor of an operation. Note that this model predicted participants’ intentions quite well with a pseudo R^2^ of 73%.

**Figure 4:**
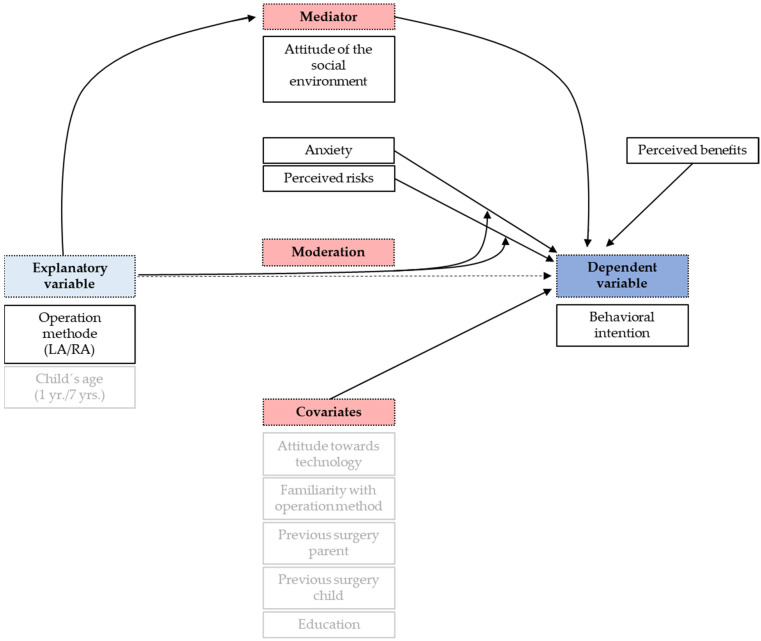
Summary of findings. The effect of operation method on behavioral intention is mediated by the attitude of the social environment. In addition, operation method moderates the influence of perceived risks and anxiety on intention. Finally, perceived benefits directly influence the behavioral intention. None of the covariates seemed to directly affect intention, although they were related to the four main predictors, which is not shown here (see Table 3, Models 3a–d).

The results shown in Figure 3 indicate that, in addition to mediation, there might also be moderation. Therefore, we conducted a moderation analysis and found significant interactions between the operation method and anxiety (*p* = 0.0015) and the operation method and perceived risk (*p* = 0.0011), while the other two interactions were not significant. Interestingly, anxiety affected parents’ intention only when facing RA surgery. The higher the anxiety level, the less likely parents would be to agree to let their child be operated on via RA surgery. Regarding the interaction of the operation method and the perceived risks, there was again a distinct statistical connection for only RA surgery. The higher the perception of risks, the lower the parents’ intention to approve RA surgery. All findings are summarized in Figure 4.

## 4. Discussion

In this first study of its kind, we examined parents‘ attitude towards robot-assisted (RA) surgery for their child. To find out which factors influence parents’ intention to let their child undergo RA, we inquired about demographic variables, attitude towards technology in general, and familiarity with different surgical procedures. In addition, we confronted them with a case scenario of pyeloplasty, in which we inquired about the perceived benefits and risks, experienced anxiety, and assumed attitude of other relevant people for both RA and LA surgery. The results were very interesting. Firstly, the affected child’s age only had little impact. A younger child (1-year-old) caused higher anxiety levels but did not have significant influence on the parents’ intention. Secondly, the operation method made a significant difference. Parents had a higher intention for LA than RA surgery.

Regarding the operation method, participants were also more familiar with LA surgery, which fits with the fact that they and their children had scarcely undergone RA surgery in the past, whereas over 60% of parents had undergone other methods of surgery in the past. Interestingly the expected benefit was higher for LA surgery, but the perceived risks were equal for both methods. Ahmad et al. [27] found a similar result, with 48% of their participants fearing higher risks with RA surgery and 42% with LA surgery. The perceived benefit was a strong predictor of parents’ intentions, which is in accordance with various previous studies [18,20,21,28]. The second strong predictor was the assumed attitude of the social environment. This finding fits well with results from various studies in other contexts (e.g., vaccination) [19,29,30,31]. Interestingly, the effect of the operation method on parents’ intention was completely mediated by the assumed attitude of others. This indicates that parents’ attitude towards RA surgery is strongly affected by what they feel their environment would favor. This issue should be addressed in future trials. It might be that the effect is attenuated if parents become more familiar with the operation method. As the “social environment” also includes medical staff, doctors must be aware of the powerful influence they have on parents. Especially in the light of surgeons offering RA surgery being perceived as more skilled, they must act with special caution [15].

The level of anxiety and perceived risk were two factors moderated by the effect of the operation method, although they were only significant for RA surgery. Additionally, parents had higher anxiety levels when encountering RA surgery, and women had higher anxiety level than men. This finding is in accordance with the international literature [32]. Anxiety was furthermore strongly related to having a child that had previously undergone surgery. While the level of expected risks is equal for LA and RA surgery, the perception of risks leads to reduced willingness to consent to RA surgery. Thus, high anxiety and risk perception levels make refusal to planned RA operation more likely. Brown et al. [33] suggested high anxiety levels among patients undergoing “new” surgical methods. Moreover, other groups have found declining readiness for RA surgery in patients with rising anxiety [21]. These findings indicate that it may make sense to inquire about anxious feelings when talking to parents.

Participants who have a positive attitude towards technology perceived more benefits and less risks. Additionally, they expected others to be more in favor of an operation. This means that parents who are skeptical about technical advances might have greater necessity to talk about risks and need more guidance and time to approach the procedure.

In the light of consent decision-making, the findings of this study require the surgeon to elaborate information provision, pointing out risks and benefits and assuring on safety of the procedure [34]. The need for more information was also emphasized in the written comments by participants. Many parents stated that they desire detailed and unbiased information about RA surgery compared to conventional laparoscopy prior to an operation. As the operation method is the decisive factor on whether anxiety has an impact on parents’ decision, fears and uncertainties should be addressed above all. With a new and relatively unknown method comes uncertainties regarding its functioning and possible malfunctioning. In a procedure often misunderstood as self-acting, the role of the surgeon should also be addressed [15,17]. In Germany, RA surgery is reimbursed by health insurances, so operation costs play no role in the decision-making. However, this might be an important issue in other countries. The surgeon should therefore address fears of high operation costs as well as fears regarding the operation method. Furthermore, it is advisable to put more emphasis on educational work on the functioning of RA surgery to minimize anxiety of a “new” and unknown procedure and enable parents to come to a responsible decision for their child.

## Figures and Tables

**Table 1:** Demographic data of the sample, listed both separately for clinical and online settings and for the whole sample.

Variable		Clinic (*n* = 92)	Online (*n* = 95)	Total (*n* = 187)
Age		M = 38.5 (SD = 6.77)	M = 36.6 (SD = 8.61)	M = 37.5 (SD = 7.82)
Number of children		M = 2.06 (SD = 0.98)	M = 1.87 (SD = 1.31)	M = 1.96 (SD = 1.16)
Gender	Female	72 (78.3%)	45 (47.4%)	117 (62.6%)
	Male	18 (19.6%)	50 (52.6%)	68 (36.4%)
	Divers	1 (1.1%)	-	1 (0.5%)
	Not given	1 (1.1%)	-	1 (0.5%)
Highest graduation	No graduation (yet)	-	1 (1.1%)	1 (0.5%)
	Secondary modern school qualification	4 (4.3%)	-	4 (2.1%)
	Higher school diploma	31 (33.7%)	3 (3.1%)	34 (18.2%)
	High school diploma	24 (26.1%)	21 (22.1%)	45 (24.1%)
	University degree	29 (31.5%)	70 (73.7%)	99 (53%)
	Not given	4 (4.4%)	-	4 (2.1%)
Previous surgery parent	Yes	83 (90.2%)	56 (59.9%)	139 (74.4%)
	No	8 (8.7%)	39 (41.1%)	47 (25.1%)
	Not given	1 (1.1%)	-	1 (0.5%)
	Under general anesthesia	76 (82.6%)	48 (50.5)	124 (66.3%)
	RA	1 (1.3%)	2 (2.1%)	3 (1.6%)
Previous surgery child	Yes	55 (59.8%)	16 (16.8%)	71 (38%)
	No	36 (39.1%)	79 (83.2%)	115 (61.5%)
	Not given	1 (1.1%)	-	1 (0.5%)
	Under general anesthesia	54 (58.7%)	13 (13.7%)	67 (35.8)
	RA	14 (15.2%)	-	14 (7.5%)
RA surgery planned	Yes	3 (3.3%)	1 (1.1%)	4 (2.1%)
	No	74 (80.4%)	83 (87.4%)	157 (84%)
	I don’t know	14 (15.2%)	11 (11.5%)	25 (13.4%)
	Not given	1 (1.1%)	-	1 (0.5%)
Attitude towards technology in general	Rating 1–5	Mean = 3.91 (SD = 0.54)	Mean = 4.18 (SD = 0.65)	Mean = 4.05 (SD = 0.61)

**Table 2:** Results on robot-assisted vs. laparoscopic surgery, means (and SD). Participating parents judged both types of surgery with respect to the same case (*n* = 187).

Variable	Robot-Assisted Surgery	Laparoscopic Surgery	Significance
Emotional reaction: anxious	6.33 (2.89)	5.96 (2.96)	*p* = 0.03
Perceived benefit	3.93 (0.65)	4.09 (0.64)	*p* = 0.006 *
Perceived risk	2.52 (0.79)	2.52 (0.70)	*p* = 0.99
Assumed attitude of social environment	2.95 (1.31)	3.45 (1.16)	*p* < 0.001 *
Behavioral Intention	3.63 (0.96)	3.97 (0.74)	*p* < 0.001 *

* significant controlling for multiple testing.

**Table 3:** Results of the models computed for the mediation analysis. Numbers show model coefficients and their significance. For each model, pseudo R^2^ (Cox and Snell) are given as well as the significance of the model compared to a null model.

	Model 1 ^a^	Model 2	Model 3a	Model 3b	Model 3c	Model 3d	Model 4
Predicted Variable	Behavioral Intention	Behavioral Intention	Anxiety	Perceived Benefits	Perceived Risks	Attitude of Social Environment	Behavioral Intention
Predictors							
Familiarity	0.17 (*p* = 0.001) *	0.13 (*p* = 0.007)	−0.06 (*p* = 0.253)	0.11 (*p* = 0.041)	−0.10 (*p* = 0.074)	0.08 (*p* = 0.125)	0.05 (*p* = 0.112)
Attitude technology	0.23 (*p* < 0.001) *	0.23 (*p* < 0.001) *	0.03 (*p* = 0.541)	.23 (*p* < 0.001) *	−0.18 (*p* = 0.001) *	0.25 (*p* < 0.001) *	0.01 (*p* = 0.654)
Previous Operation Child	−0.24 (*p* = 0.025)	−0.23 (*p* = 0.024)	0.49 (*p* < 0.001) *	−0.25 (*p* = 0.021)	0.29 (*p* = 0.015)	−0.21 (*p* < 0.001) *	0.02 (*p* = 0.762)
Operation method (RA vs. LA)		−0.34 (*p* < 0.001)*	0.11 (*p* = 0.122)	−0.21 (*p* = 0.025)	−0.05 (*p* = 0.640)	−0.44 (*p* < 0.001) *	−0.07 (*p* = 0.281)
Age of child (7 vs. 1 years)		0.09 (*p* = 0.338)	−0.21 (*p* = 0.054)	0.10 (*p* = 0.331)	−0.05 (*p* = 0.668)	−0.03 (*p* = 0.803)	0.05 (*p* = 0.407)
Anxiety							−0.13 (*p* < 0.001) *
Perceived benefits							0.42 (*p* < 0.001) *
Perceived risks							−0.04 (*p* = 0.224)
Attitude of social environment							0.43 (*p* < 0.001) *
Pseudo R^2^	0.11	0.14	0.08	0.11	0.07	0.15	0.73
(Cox and Snell)							
Significance Model	*p* < 0.001	*p* < 0.001	*p* < 0.001	*p* < 0.001	*p* < 0.001	*p* < 0.001	*p* < 0.001

Notes: ^a^ All other covariates did not turn out to be significant predictors of behavioral intention and were therefore not included in this and the other models, * significant controlling for the number of tests.

## Data Availability

The data presented in this study are available on request from the corresponding author. The data are not publicly available due to an internal policy not to make any patient-related data publicly available.

## Appendix A. Questionnaire in English Translation

**Questionnaire**

Please complete the questions at your personal discretion and without the help of others. We look forward to receiving your responses. For each of the following statements, indicate how strongly you agree or disagree.

*Please select only one answer for each question.*
**Questions about robot-assisted surgery**

As a reminder, robot-assisted surgery is the assisted use of a robot during surgery. It is a minimally invasive procedure as a camera and the necessary instruments are inserted into the patient’s abdomen through small skin incisions. The physician sits at a control device and steers the arms of the surgical robot from there. In this way, the hand and finger movements are transferred directly to the surgical instruments and executed by the robotic arms. The image is transmitted to a monitor via the camera.

	**Do Not Agree at All**	**Do Not Agree**	**Neither**	**Agree**	**Fully Agree**
I am familiar with robot-assisted surgery.					
I have a lot of knowledge about robot-assisted surgery					
I know more about robot-assisted surgery than the average person.					

**Technology questions**

	**Do Not Agree at All**	**Do Not Agree**	**Neither**	**Agree**	**Fully Agree**
Technical progress is good for mankind.					
Technology enables people to live comfortably.					
Technology is more of a threat than an advantage to people.					
Technology restricts people in their personal freedom.					
Technical devices are often opaque and difficult for me to control.					
I like to try out new technical devices.					

**Questions about laparoscopic surgery**

As a reminder, laparoscopy is a minimally invasive surgical procedure because it does not require large incisions. A camera and the necessary instruments are inserted into the patient’s abdomen through small skin incisions (“keyhole technique”). The camera transmits the image to a monitor. The surgeon thus moves the surgical instruments directly on the patient.

	**Do Not Agree at All**	**Do Not Agree**	**Neither**	**Agree**	**Fully Agree**
I am familiar with laparoscopic surgery.					
I have a lot of knowledge about laparoscopic surgery.					
I know more about laparoscopic surgery than the average person.					

**Scenario part 1**

Imagine you have a 1-year-old child who has been diagnosed with kidney disease during a routine hospital examination. In this case, the transition from the kidney to the ureter is narrowed, so the outflow of urine is impaired and the kidney is already significantly affected as a result. Without surgical correction of this constriction, the kidney is likely to lose all or part of its function. In this hospital, there is an option of performing the surgery either robot-assisted or laparoscopically.

In this case, the doctors advise you to undergo laparoscopic surgery. In laparoscopic surgery (“keyhole technique”), the operation is performed by a doctor himself. Instruments and a video camera are inserted into the patient’s body through several small openings. The surgeon (in the picture on the left) thus moves the surgical instruments directly in the patient.

The doctors point out that—as with any operation—pain and a hospital stay of a few days are to be expected. In addition, complications may arise that may result in a follow-up operation (for example, injury to neighboring organs during the operation).

Here, you can see a sketch of the operating room:

**Please put yourself in the position described as best you can and answer the following questions regarding your 1-year-old’s laparoscopic surgery for ureteral stenosis.**

For each of the following statements, indicate how strongly you agree or disagree. Please select only one answer for each question.

	**0** **Not at All**	**1**	**2**	**3**	**4**	**5**	**6**	**7**	**8**	**9**	**10** **Very Strong**
I feel angry.											
I feel anxious.											
I feel happy.											
I feel disgusted.											
I feel surprised.											
I feel sad.											

	**Do Not Agree at All**	**Do Not Agree**	**Neither**	**Agree**	**Fully Agree**
In this situation, I would expect long-term negative consequences for my child (e.g., irreversible damage, follow-up surgery).					
In this situation, I would be willing to let my child undergo laparoscopic surgery as recommended.					
In this situation, I would expect an overall good surgical outcome.					
In this situation, I would expect short-term negative consequences for my child (e.g., pain, longer than average surgery time).					
In this situation, I would feel safe to have my child operated on laparoscopically as recommended.					
In this situation, the recommended laparoscopic procedure would make sense for my child.					
In this situation, I would expect technical problems during the operation.					
In this situation, I would expect a complete solution to the problem.					
In this situation, I would like to have my child operated on laparoscopically as recommended.					

	**Do Not Agree at All**	**Do Not Agree**	**Neither**	**Agree**	**Fully Agree**	**Don’t Know**
My family would advise me to have the surgery laparoscopically as recommended.						
My friends would advise me to have the surgery laparoscopically as recommended.						
Other physicians would advise me to have the surgery laparoscopically as recommended.						

**Scenario part 2**

Imagine you want to get a second opinion regarding the diagnosis and the surgical procedure. To do this, you go to a nearby hospital to have your 1-year-old child examined again. The doctors there agree with the diagnosis of a narrowed ureter as already made in the previous hospital. Again, there are options of robot-assisted surgery and laparoscopic surgery.

In this hospital, the doctors advise you to undergo surgery with a surgical robot. In robot-assisted surgery, the doctor sits in the operating room at a control device (pictured left) and steers the arms of a surgical robot via a console. Instruments and a video camera are inserted into the patient’s body through several small openings. The physician’s hand and finger movements are transferred to the surgical instruments and executed by the robotic arms (pictured right).

The doctors point out that—as with any operation—pain and a hospital stay of a few days are to be expected. In addition, complications may arise that may result in a follow-up operation (for example, injury to neighboring organs during the operation).

Here, you can see a sketch of the operating room:

**Please put yourself in the position described as best you can and answer the following questions regarding your 1-year-old’s robotic-assisted surgery for ureteral stenosis.**

For each of the following statements, indicate how strongly you agree or disagree. Please select only one answer for each question.

	**0** **Not at All**	**1**	**2**	**3**	**4**	**5**	**6**	**7**	**8**	**9**	**10** **Very Strong**
I feel surprised.											
I feel anxious.											
I feel disgusted.											
I feel angry.											
I feel sad.											
I feel happy.											

	**Do Not Agree at All**	**Do Not Agree**	**Neither**	**Agree**	**Fully Agree**
In this situation, I would expect a complete solution to the problem.					
In this situation, I would be willing to let my child undergo robot-assisted surgery as recommended.					
In this situation, I would feel safe to let my child undergo robot-assisted surgery as recommended.					
In this situation, the recommended robotic-assisted surgery would be useful for my child.					
In this situation, I would expect long-term negative consequences for my child (e.g., irreversible damage, follow-up surgery).					
In this situation, I would like to have my child undergo robot-assisted surgery as recommended.					
In this situation, I would expect short-term negative consequences for my child (e.g., pain, longer than average surgery time).					
In this situation, I would expect technical problems during the operation.					
In this situation, I would expect an overall good surgical outcome.					

	**Do Not Agree at All**	**Do Not Agree**	**Neither**	**Agree**	**Fully Agree**	**Don’t Know**
My friends would advise me to have the surgery robot-assisted as recommended.						
My family would advise me to have the surgery robot-assisted as recommended.						
Other physicians would advise me to have the surgery robot-assisted as recommended.						

**Questionnaire**

*The following questions are about you*.						
**Gender**	male	****	female	****	diverse	****
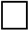
**Age** __________						
**Number of children** ______________						
**Highest school degree**						
****	No school-leaving qualification (yet)					
	Secondary school diploma					
****	Secondary school leaving certificate					
	Abitur (general university entrance qualification), subject-related university entrance qualification or technical college entrance qualification					
****	University degree (Bachelor, Master, Magister, Diploma, State Examination, Doctorate)					
	Other: ________________________________________________					
**Have you ever had surgery?**			Yes	****	No	
If yes: Under general anaesthesia?			Yes	****	No	
If yes: Robot-assisted?			Yes	****	No	
**Have any of your children ever had surgery?**			Yes	****	No	
If yes: Under general anaesthesia?			Yes	****	No	
If yes: Robot-assisted?			Yes	****	No	
**Is a robot-assisted surgical procedure planned for any of your children in the near future?**						
	Yes	****	No		Don’t know	****
**If you are or actually were in the situation described in the scenarios, what additional information would you have wanted in the decision-making process?**						
**_____________________________________________________________________________________**						
**_____________________________________________________________________________________**						
**_____________________________________________________________________________________**						
**Thank you for your participation!**						
